# Tumor ecosystem subtyping of breast cancer based on somatic mutations and network propagation reveals distinct prognostic and genomic landscapes

**DOI:** 10.3389/fgene.2026.1847952

**Published:** 2026-05-15

**Authors:** Ke Ding, Zixuan Zhu, Zhengchun Huo, Honghao Li, Haoran Chen, Shiyuan Wang, Lei Yang

**Affiliations:** 1 The First Affiliated Hospital of Harbin Medical University, School of Stomatology, Harbin Medical University, Harbin, China; 2 College of Bioinformatics Science and Technology, Harbin Medical University, Harbin, China

**Keywords:** breast cancer, immunotherapy response, machine learning, network propagation, somatic mutation profile, tumor ecosystem

## Abstract

**Introduction:**

Breast cancer is the most common malignancy in women worldwide, exhibiting high heterogeneity that complicates diagnosis, treatment, and prognosis. While somatic mutations stably reveal genetic characteristics of tumor cells, their application in breast cancer subtyping remains underexplored.

**Methods:**

A total of 2,526 breast cancer patients from Memorial Sloan Kettering Cancer Center were classified into different tumor ecosystem subtypes (TESs) based on somatic mutation profiles using a network propagation algorithm.

**Results:**

The prognosis of breast cancer patients in TES 1 was significantly better than that of those in TES 2. Immunological characterization further revealed that the tumor microenvironment contained significantly more tumor immune cells in TES 1 than in TES 2, and that TES 2 had lower response to immunotherapy but was more sensitive to chemotherapeutic agents. Moreover, our tumor ecosystem subtyping method effectively classified patients across 20 cancer cohorts with good generalization.

**Conclusion:**

This study proposes a stable, reproducible, and clinically applicable subtyping strategy based on somatic mutation data for tumor ecosystem subtyping, which can be used to guide personalized treatment for breast cancer patients and promote the development of precision medicine.

## 1 Introduction

In recent years, breast cancer is among the most frequently diagnosed malignancies globally, accounting for 11.6% of all cancers worldwide and 6.9% of all cancer deaths in 2022 (Bray et al., 2024). Breast cancer is also one of the most widespread cancers in women, with 316,950 new cases estimated in the United States in 2025, accounting for 32% of all female cancers (Siegel et al., 2025). Recent advances in breast cancer prevention and treatment technologies, including surgical intervention, radiotherapy, chemotherapy, and endocrine therapy, have markedly improved the prognosis of breast cancer patients (Kerr et al., 2022). Breast cancer survival rates have improved significantly in recent decades, primarily due to advances in early detection and treatment. According to 2025 statistics, the 5-year relative survival rate for breast cancer can reach 99% if diagnosed at the local stage, while it drops to approximately 30% for metastatic cases (Giaquinto et al., 2024).

Breast cancer is characterized by significant heterogeneity, which complicates early diagnosis, treatment, and prognosis, leading to numerous clinical challenges in breast cancer management (Saleem et al., 2020). In 2000, Perou et al. introduced the concept of molecular subtyping of breast cancer, highlighting tumor heterogeneity based on gene expression profile (Perou et al., 2000). Subsequently, leveraging large-scale genomic and transcriptome data, scholars subdivided breast cancer into multiple molecular subtypes correlated these subtypes with clinical prognosis to inform targeted therapy and chemotherapy decisions (METABRIC et al., 2012; Lehmann et al., 2016; Chen et al., 2025; Xiong et al., 2025). The 13th St. Gallen International Breast Cancer Conference (2013) emphasized the centrality of the molecular subtyping of breast cancer in treatment decisions (Goldhirsch et al., 2013).

In contemporary breast cancer research, multi-omics data is widely utilized to stratify patients into distinct molecular subtypes (Xiao et al., 2022; Sørlie et al., 2001). However, somatic mutation data are seldom employed for breast cancer classification (Hou et al., 2021, p. 1; Chen et al., 2023; Chen et al., 2025; Zhang et al., 2025), largely in light of their inherent sparsity, lack of standardization for analysis, and relatively limited practical applicability (Jiang et al., 2024). Therefore, it is imperative to develop hierarchical algorithms for somatic mutation to automate and standardize the analysis process of somatic mutation data (Dimakopoulos et al., 2007).

Cancer progression is typically driven by the accumulation of somatic gene mutations, which can directly reflect the genetic characteristics of tumors. Consequently, the classification of cancer patients based on somatic mutation data can facilitate the identification of cancer subtypes. To address the challenges posed by the sparse somatic mutation data, various algorithms have been proposed to optimize mutations spectrum and classify cancer patients into different subtypes (Chuang et al., 2007; Hristov and Singh, 2017; Rohani and Eslahchi, 2020; Luo et al., 2025; Su et al., 2025).

In this study, we combined the somatic mutation spectrum of breast cancer patients with the network propagation algorithm to promote statistical analysis and enhance the utility of mutation data. We utilized the somatic mutation profile exclusively from breast cancer patients at Memorial Sloan Kettering Cancer Center (MSKCC) (Cheng et al., 2015) to classify patients into two TESs with different prognoses. The network propagation algorithm was employed to convert the original somatic cell binary vector data, indicating the presence or absence of mutations, into continuous numerical vectors to smooth the sparse mutation data and make it suitable for subsequent analysis. Subsequently, ssGSEA was performed using a network propagation-based somatic mutation spectrum (Hänzelmann et al., 2013) and the enrichment scores of 68 genesets (Wu R. et al., 2023) depicting the breast cancer ecosystem were assessed for each breast cancer patient. Then, based on the enrichment scores of genesets, MSKCC breast cancer patients were stratified into two TESs using the K-means algorithm. These two TESs exhibited significant differences in prognosis, immunological characteristics, and therapeutic effects. Our findings illustrate the feasibility of identifying TESs of breast cancer solely from somatic mutation data. Collectively, this work evaluates the feasibility of identifying breast cancer ecosystem states using somatic mutation data alone and positions this framework as a complementary, hypothesis-generating stratification strategy for precision oncology research.

## 2 Materials and methods

### 2.1 Public data collection

The breast cancer cohort was derived from the work of Nguyen et al. (Nguyen et al., 2022), with its somatic mutation profiles, genomic data, and clinical data. The research aggregated a massive clinico-genomic cohort from MSKCC that included over 25,000 metastatic cases across 50 tumor types. Between 18 November 2013 and 18 August 2021, these cancer cases were sequenced at MSKCC.

Notably, these samples represent 462 mutation genes and 2,526 unique patients of breast cancer sequenced at MSKCC from 18 November 2013 to 18 August 2021. Simultaneously, there were other mutation data including bladder urothelial carcinoma (1,141 samples), colorectal adenocarcinoma (3,537 samples), pancreatic adenocarcinoma (1942 samples), prostate adenocarcinoma (1951 samples), etc. The 2,526 breast cancer samples were divided into training and testing cohorts in a 7:3 ratio. The training cohort comprised 1768 samples, while the testing cohort consisted of 758 samples.

We collected 68 genesets depicting the breast cancer ecosystem, including the genesets for inherent properties of tumor cells such as proliferation, epithelial-to-mesenchymal transition (EMT), inflammation, etc., the genesets encompassing markers of cells in the microenvironment such as T cells, NK cells, etc. (Wu et al., 2021). These gene sets were used as predefined functional annotations to summarize mutation-derived signals at the ecosystem level.

### 2.2 Genome-scale protein network

Human protein-protein interactions were retrieved from the STRING database (version 11.5) (Szklarczyk et al., 2019). To guarantee the use of reliable human protein-protein interactions, only links with interaction scores greater than 700 were selected for further analysis. Consequently, we obtained 474,722 protein interaction pairs, derived from 16,134 genes.

### 2.3 Network propagation for mutant profile

To make the mutation profile more suitable for statistical analysis, we adopted a method that incorporates the somatic mutation profile with network propagation to generate a propagation mutation profile. The random walk with restart (RWR) is defined as an iterative process whereby a walker transitions from its initial node (or current node) to a randomly selected neighbor in close proximity, on the basis of the connecting edge probabilities. A restart probability r is used to control the probability that the random walk returns to the starting state whose value is 0.75 in this study. Let Po be the starting probability distribution vector, which represents the starting state where only the starting node (somatic mutated genes specific to each breast cancer sample) has a non-zero probability ranging from 0 to 1, while the other genes were set a value of zero. The iterative form of the probability distribution at step t + 1 can be calculated as Equation 1:

$$p_{t+1}=\left(1-r\right)Wp_{t}+rp_{0}$$ (1)

where W is the Transition matrix for graph and $p_{t}$ represents the probability distribution of each node being accessed during the random walk at step t. Updates were stopped when $\|p_{t+1}-p_{t}\left\|<\right.10^{-6}$ , and then all candidate nodes were ranked by the steady-state probability vector p∞ .

The algorithm utilizes network topology, edge weights, and long-range interactions between proteins. This study used the RWR algorithm from the R package dnet (version 1.1.7) to propagate the network. The application of the network propagation algorithm resulted in a breast cancer gene interaction network, which included a substantial number of gene nodes and interaction edges between them. This network facilitates the identification of key gene nodes and important interaction pathways.

### 2.4 Gene set variation analysis

The ssGSEA, implemented in the R package GSVA (version 1.48.1), was employed to calculate the normalized enrichment scores (NES) of 68 genesets in the network propagation mutation profile. For both the training cohort and testing cohort, we enriched the gene mutation profile into the tumor ecosystem through GSVA and acquired the NES of each sample.

### 2.5 Sample clustering and prognostic analysis

To identify which genesets significantly demonstrated associations with overall survival, a univariate Cox regression analysis was performed on the 68 genesets in the training cohort. The univariate Cox regression was conducted utilizing the R package survival software (version 3.2-11). We acquired Cox results and extracted 62 genesets whose P-values were less than the threshold (P-value<0.05), which represents genesets that were significantly associated with survival time.

Next, the K-means algorithm was utilized to categorize patients into distinct TES in the training and testing cohort. We calculated the average silhouette width to assess the most suitable cluster. Subsequently, the survival analysis was performed using the R package survival.

## 3 Results

### 3.1 An overview of the mutational landscape of breast cancer patients

An exhaustive depiction of 12,128 somatic variations, across three distinct MSK-IMPACT gene panels, was achieved for a comprehensive set of 2,526 breast cancer specimens utilizing the maftools R package (version 2.8.05). We constructed a mutation-counting matrix devoid of synonymous mutations to record the genetic variation information in each sample, using the mutCountMatrix function from the R package maftools. These variants included single nucleotide polymorphisms (SNPs), insertion/deletion variants (indel), etc. Any samples or genes that did not have mutations were removed from the result matrix. Ultimately, a mutation-counting matrix, encompassing 462 distinct mutated genes across 2,526 individual samples was derived.

### 3.2 Recognition and further analyses of TESs associated with prognosis

The univariate Cox proportional risk model was analyzed in the training cohort, and the 62 genesets with P-values less than 0.05 were saved. There were 47 genesets, including apoptosis, cell cycle, DNA damage, Hypoxia, HYPOXIA, DNA REPAIR, etc., indicating that they may be associated with poor prognosis. 15 genesets, including effector cells, NK cells, T cells, B cells, ANGIOGENESIS, etc., were associated with good prognosis.

**FIGURE 1:**
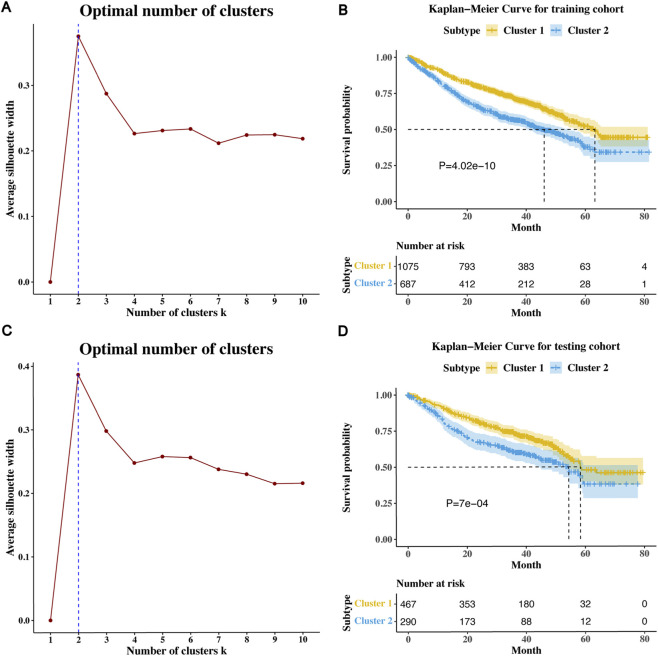
Classification and analysis of TESs related to prognosis. **(A)** Average silhouette coefficient of the training cohort. **(B)** Kaplan-Meier curves of overall survival for two TESs in training cohort. **(C)** Average silhouette coefficient of the testing cohort. **(D)** Kaplan-Meier curves of overall survival for two TESs in testing cohort.

The training cohort encompassed a total of 1768 breast cancer patients with suitably matched overall survival data and clinicopathological data to validate the cluster subtype model. Employing the 62 genesets discerned from the training cohort, we applied the K-means clustering algorithm to stratification TES for each patient. Through an exhaustive examination of k values ranging from 2 to 10, the optimal number of clusters was determined to be k = 2, yielding the highest average silhouette coefficient (Figure 1A). The Kaplan-Meier survival curve showed that breast cancer patients belonging to TES 2 had significantly lower overall survival compared with breast cancer patients belonging to TES 1 in the training cohort (P-value <0.05; Log-rank test; Figure 1B). Additionally, the optimal number of clusters obtained in the testing cohort was also 2 (Figure 1C), and the Kaplan-Meier survival curve exhibited a comparable trend to that observed in the testing cohort (Figure 1D).

**FIGURE 2:**
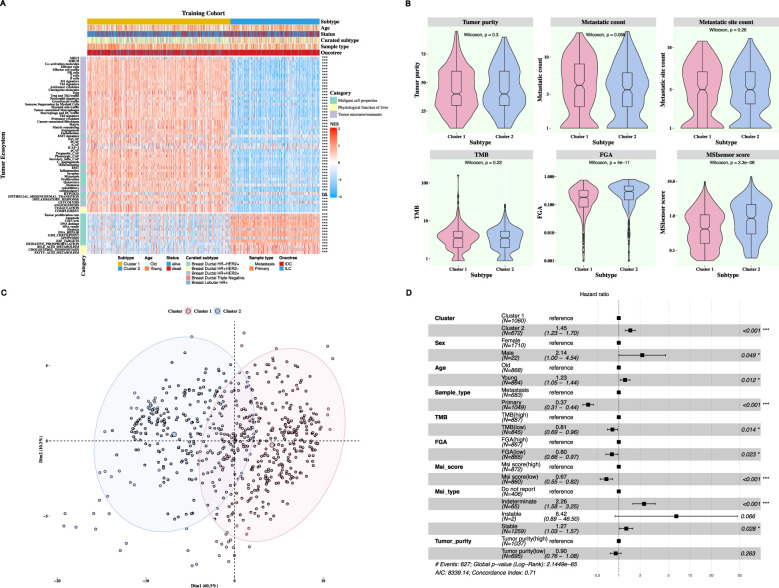
The clinical signature and functional characteristics of two TESs in the training cohort. **(A)** The distribution of NES levels across 68 genesets discerning variations between two TESs. (ns indicated P-value>0.05; *indicated P-value<0.05; **indicated P-value<0.01; ***indicated P-value<0.001; Wilcoxon rank-sum test). **(B)** The violin plots distribution of tumor purity, metastatic count, metastatic site count, TMB, FGA and MSIsensor score between two TESs. **(C)** The PCA analysis projected breast cancer samples onto two-dimensional spatial coordinates, demonstrating good discrimination for two TESs. **(D)** A forest plot of multivariate Cox regression analysis of the TESs and clinical annotations for breast cancer patients.

To assess and contract the tumor ecosystem of two TESs in the training cohort, the NES levels of 68 genesets depicting the breast cancer ecosystem were compared between two TESs. Wilcoxon rank sum test revealed that there were statistically significant differences in NES levels of 67 genesets between two TESs in the training cohort (Figure 2A). The NES levels of 53 genesets of tumor ecosystem in TES 1, such as MHCI cells, MHCII cells, effector cells, Effector cell traffic cells, NK cells, T cells, B cells, Treg cells, M1 cells, were higher than those of patients with TES 2. This may be attributed to the ability of these immune cells to enhance the recognition and elimination of tumors by amplifying the anti-tumor immune response and regulating the immune microenvironment. For instance, dendritic cell (DC) activate T cells through antigen presentation, while neutrophils directly kill tumor cells by releasing cytotoxic particles. Furthermore, highly expressed MHC-I molecules can enhance tumor cells' recognition of CD8^+^ cytotoxic T cells, thus promoting immune surveillance (Wu X. et al., 2023). In addition, the high expression of MHC-II molecules has been linked to an increase in tumor-invasive T cells, which, in turn, activates CD4^+^ helper T cells, thereby boosting anti-tumor immunity (Macy et al., 2023). Moreover, B cells can play an anti-tumor role in breast cancer through various mechanisms such as direct killing of tumor cells, antigen presentation, secretion of antibodies and cytokines (Qin et al., 2021). On the other hand, the levels of 14 genesets of tumor ecosystem in TES 2, such as tumor proliferation rate, apoptosis, cell cycle, DNA damage, exceeded those of patients with TES 1. Among them, hypoxia is one of the characteristics of tumor microenvironment. Tumor cells promote angiogenesis and metabolic reprogramming by activating the hypoxia-inducing factor (HIF) pathway. In addition, hypoxia can also inhibit the function of immune cells, thus promoting the evasion of tumor immunity (Emami Nejad et al., 2021). Breast cancer patients with a poor prognosis frequently demonstrate an elevated rate of tumor cell proliferation, a state which is correlated with dysregulation of the cell cycle. For example, high expression of E2F target genes can not only facilitate the progression of cell cycle (G1/S and G2/M phases) and enable rapid proliferation of tumor cells but also enhance drug resistance of tumor cells by regulating signaling pathways in the tumor microenvironment. Similar results were found in the testing cohort (Supplementary Figure S1A).

In addition, metastatic site count, TMB, FGA, and MSIsensor score were statistically significant differences between the two TESs (P-value <0.05; Wilcoxon rank-sum test; Figure 2B). While there is no statistically significant difference in tumor purity and metastatic count between two TESs (P-value>0.05; Wilcoxon rank-sum test). In the testing cohort, there were significant differences in the FGA and MSIsensor score between two TESs (P-value<0.05; Wilcoxon rank-sum test; Supplementary Figure S1B).

Moreover, PCA revealed a distinct separation between two TESs based on NES scores (Figure 2C). Similar results were found in the testing cohort (Supplementary Figure S1C). Further multivariate Cox regression analysis exhibited that cluster, sex, age, tumor metastasis type, TMB, FGA, MSIsensor score, and MSIsensor type emerged as independent predictors of overall survival, thereby underscoring their pivotal role in prognostic assessment (P-value<0.05; Figure 2D). Whereas in the testing cohort, only sex, age, tumor metastasis type, and MSIsensor score were independent predictors (Supplementary Figure S1D).

### 3.3 Enrichment analysis of two TESs

**FIGURE 3:**
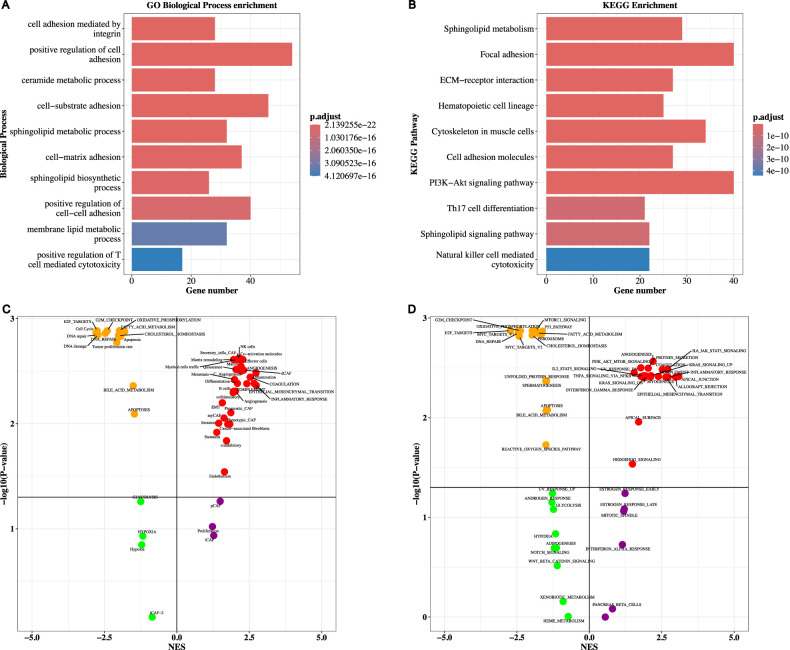
Enrichment analysis result of two TESs in training cohort. **(A)** GO biological process terms and **(B)** KEGG pathways for the differential genes between two TESs. **(C)** Volcano plot illustrated the GSEA results of 68 genesets and **(D)** 50 Halllmarks between two TESs.

To explore the molecular mechanism and clinically relevant molecular targets of breast cancer, the network propagation-based gene profile of 16134 genes derived from the somatic mutation profile between two TESs in the training cohort was compared. A total of 411 differential genes between two cluster subtype samples (| ${\mathrm{Log}}_{2}$ FC|>1.0 and P-value<0.05) were obtained. The enrichment analysis results for GO biological processes and KEGG pathways are respectively presented in Figures 3A,B. The testing cohort demonstrated comparable results (Supplementary Figure S2A,B).

To delve into the fundamental mechanisms underlying the distinct prognostic disparities observed among the two distinct cluster subtypes, we performed a GSEA, leveraging 68 tumor ecosystem genesets and 50 Hallmarks. For 68 tumor ecosystem genesets, as illustrated in Figure 3C and Supplementary Figure S3A, TES 1 displayed significant enrichment in 30 genesets. While the TES 2 illustrated significant enrichment in 13 genesets (Figure 3C). Similar results were obtained in the testing cohort (Supplementary Figures S2B, C). When comparing the differences between two TESs in 50 Hallmarks, TES 1 was significantly enriched in 19 Hallmarks, while TES 2 was significantly enriched in 16 Hallmarks (Figure 3D; Supplementary Figure S2C). Similar results were illustrated in the testing cohort (Supplementary Figures S2D,3D).

### 3.4 Analysis of the relationship between gene modules and sample traits

The WGCNA (Langfelder and Horvath, 2008) was conducted on the 68 genesets to identify which signatures were associated clustering subtype. Firstly, the 68 genesets of all samples were clustered, and those with the same expression pattern were clustered together. To ascertain an optimal power level for the construction of a co-expression network, we set the soft threshold as 6 (Supplementary Figure S4A), yielding a scale-free $R^{2}$ metric of 0.09 (Supplementary Figure S4B). According to the selected soft threshold, the adjacency matrix between genes was constructed and converted into topological overlap matrix (TOM). The differences between genesets were calculated, and the genesets were clustered using hierarchical clustering. A one-step method was applied to generate co-expression network and then modules were identified, so that the genesets with high NES similarity were assigned to the same module. The hierarchical clustering tree and module colors were visualized to show the distribution of modules. The hierarchical clustering algorithm identifies two modules with different colors (Supplementary Figure S4C), among which the turquoise module exhibited a notably significant correlation with the defined clustering subtype (Supplementary Figure S4D). An in-depth exploration of the turquoise module and the subtype attributes revealed a remarkable correlation coefficient of 0.97 (Supplementary Figure S4E) between significance metrics (GS) and module membership (MM) indices within the turquoise framework, thereby underscoring the exceptional quality and robustness of the turquoise module constructed herein.

### 3.5 Immunological research of two different TESs

**FIGURE 4:**
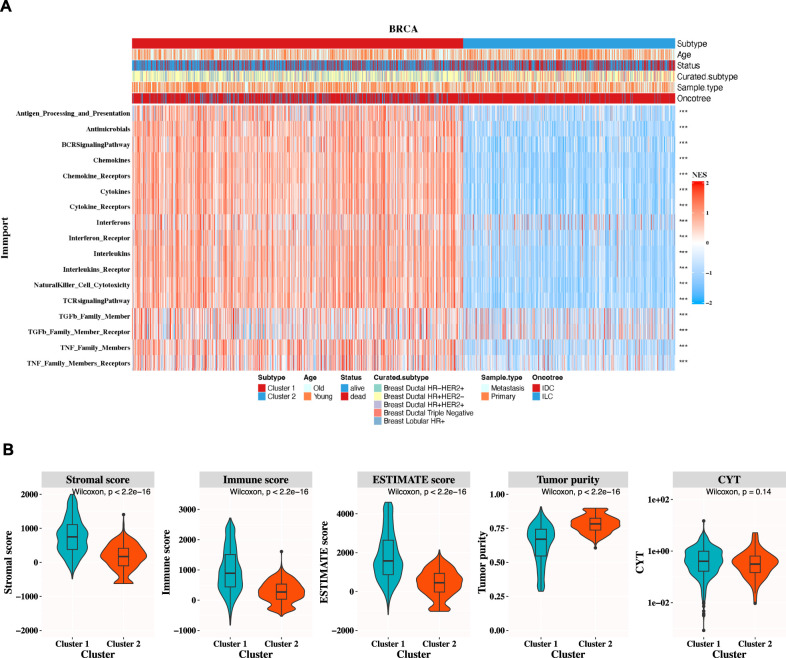
Immunological research of two different TESs in the training cohort. **(A)** The NES levels of 72 immune checkpoints in two distinct breast cancer TESs. (ICP indicated immune checkpoint; ns indicated P-value>0.05; * indicated P-value<0.05; ** indicated P-value<0.01; *** indicated P-value<0.001; Wilcoxon rank-sum test). **(B)** Violin plots illustrated the differences in stromal score, immune score, ESTIMATE score, tumor purity, and CYT of two TESs.

Immune checkpoints (ICP) have been demonstrated to play an inhibitory role in the progression of tumors. Therefore, the mRNA expression levels of immune checkpoints are deemed as essential biological markers in the field of cancer immunotherapy. To facilitate a comparison of the immune checkpoint characteristics between two TESs, 72 immune checkpoints were studied, and their NES levels were compared in two different breast cancer TESs (Figure 4A).

As depicted in Figure 4A, in comparison to patients with TES 2, majority of immune checkpoints in TES 1 exhibited elevated NES levels, suggesting that the unfavorable prognosis observed in TES 2 might be relative to the immunosuppressive microenvironment (P-value<0.05, Wilcoxon rank-sum test). Similar results were found in the testing cohort (Supplementary Figure S5A). To assess the composition of the tumor microenvironment, the ESTIMATE algorithm was utilized to estimate the stromal score, immune score, ESTIMATE score, tumor purity, and CYT. Our findings disclosed statistically noteworthy variations in tumor purity, ESTIMATE score, stromal score as well as immune score, between the two subtypes in the training cohort (P-value<0.05; Wilcoxon rank-sum test, Figure 4B). Similar results were found in the testing cohort (Supplementary Figure S5B).

### 3.6 Establishment and verification of XGBoost classifier

**FIGURE 5:**
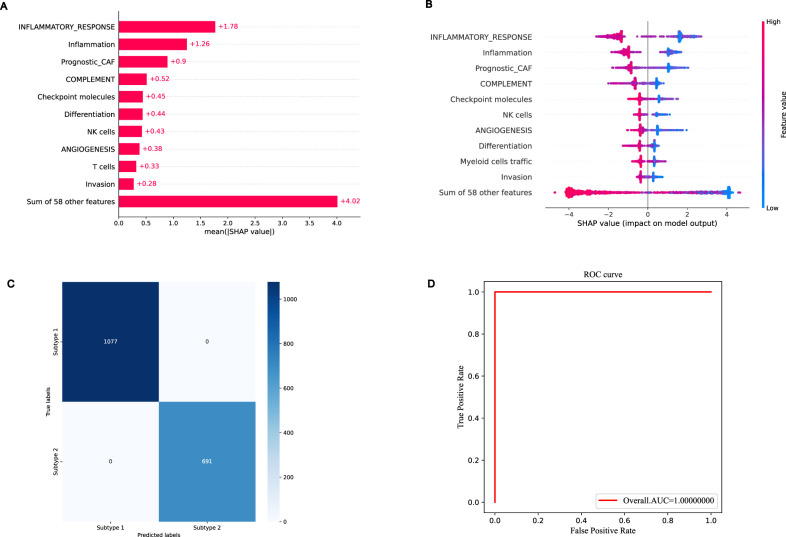
Prediction of breast cancer patients with different TESs by XGBoost algorithm in the training cohort. **(A)** The mean absolute SHAP values of the top 10 genesets. **(B)** Distribution of the SHAP values for the top 10 genesets based on the highest mean absolute SHAP value. **(C)** Confusion matrix for prediction of breast cancer patients. **(D)** The ROC curve of XGBoost model for prediction of breast cancer patients.

Utilizing the NES levels of 68 genesets, we attained a comprehensive accuracy of 98.08%, employing a rigorous 10-fold cross-validation procedure within our training cohort. The predictive outcomes indicated the aptness of the XGBoost methodology for classifying the distinct TESs of breast cancer patients, thereby reinforcing its suitability for such application (Guo et al., 2025; Li et al., 2025; Rajpoot, 2025; Momanyi et al., 2026; Riasat et al., 2026). Based on the analysis of mean absolute SHAP values and individual SHAP contributions, Figures 5A,B respectively visualize and rank the SHAP importance values for the 10 most influential tumor ecosystem genesets within the training cohort. These genesets are listed in descending order of their impact on the model’s prediction results. In the training cohort, we identified inflammatory response, inflammation, prognostic CAF, complement, checkpoint molecules, differentiation processes, NK cell activity, angiogenesis, T-cell involvement, and invasion as the primary drivers of the model’s predictive prowess. Each of these genesets contribute favorably to enhancing the predictive accuracy of the model. Similar results were found in the testing cohort (Supplementary Figures S6A,B).

Given the robust predictive capability of the XGBoost model in discerning distinct TESs among breast cancer patients, the model, tailored from the training cohort, was subsequently employed to ascertain the cluster labels of patients within the testing cohort, thereby enhancing the analysis’s rigor and adherence to scientific standards. The anticipated outcomes reveal that within the cohort of 467 breast cancer individuals belonging to TES 1, a precise categorization was achieved for 460 patients. Similarly, among the 291 patients classified under TES 2, 287 were accurately identified (Figure 5C). The estimated area under the receiver operating characteristic (ROC) curve attained a value of 1.0000 (Figure 5D). These findings indicate the proficiency of our XGBoost classifier in precisely forecasting the classification of breast cancer patients whose subtypes are currently unascertained. Similar results were found in the testing cohort (Supplementary Figure S6C), and the ROC curve obtained in the testing cohort is 0.9994 (Supplementary Figure S6D).

### 3.7 Model validation results for different cancer cohorts

**FIGURE 6:**
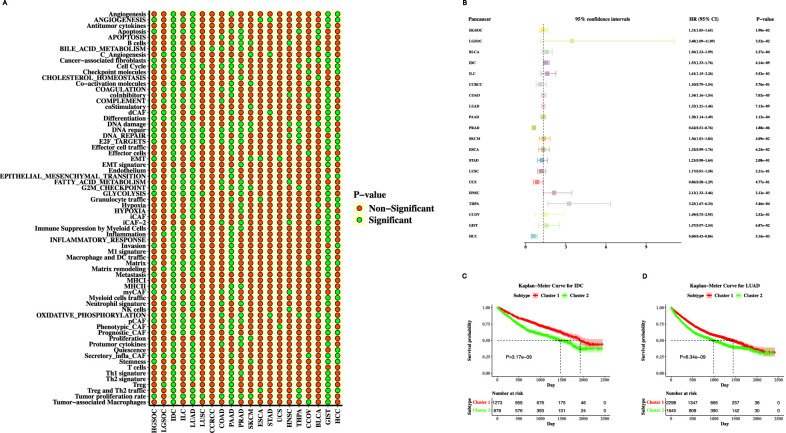
Validation of our method on 20 different MSKCC cancer types. **(A)** Bubble plot illustrated the prognostic value of 68 genesets by using a univariate Cox proportional hazards regression analysis for 20 cancer cohorts in the MSKCC database. **(B)** The forest plot illustrated the 95% CI, HR and P-value for 20 cancer types in the MSKCC database. The 95% CI, HR and P-value were determined by the univariate Cox regression analysis. Kaplan-Meier survival curves for **(C)** IDC and **(D)** LUAD.

We then validated the robustness of our model in 20 different cancer cohorts, including bladder urothelial carcinoma (BLCA), invasive ductal carcinoma (IDC), invasive lobular carcinoma (ILC), colon cancer (COAD), lung adenocarcinoma (LUAD), pancreatic cancer (PAAD), etc. The univariate Cox regression showed the correlation between the prognosis of different cancer cohorts and genesets that were significantly associated with cancer prognosis were illustrated in Figure 6A. The clustering results showed that except for thyroid papillary (THPA) and cutaneous melanoma (SKCM), the optimal number of clusters for most other cancers was two.

The Kaplan-Meier analysis of survival curves revealed a non-significant statistical disparity in outcomes among two clusters for six cancer types, including hepatocellular carcinoma (HCC), ovarian clear cell carcinoma (CCOV), renal clear cell carcinoma (CCRCC), gastrointestinal stromal tumors (GIST), and esophageal carcinoma (ESCA). This finding underscores the absence of a pronounced difference in prognosis between two TESs across these specific cancer types. However, in the other 15 cancer cohorts, there was a statistically significant difference in outcomes between TES 1 and TES 2 (P-value<0.05, Log-rank test; Figures 6B–D). These cancers include bladder urothelial carcinoma (BLCA), pancreatic adenocarcinoma (PAAD), colon adenocarcinoma (COAD), high-grade serous ovarian cancer (HGSOC), head and neck squamous cell carcinoma (HNSC), invasive ductal carcinoma (IDC), lung adenocarcinoma (LUAD), etc.

## 4 Discussion

Breast cancer is the most prevalent cancer in women worldwide, and its heterogeneity poses significant challenges for diagnosis, treatment and prevention. The molecular subtyping of breast cancer has enhanced the understanding and precision treatment of the disease, however, conventional molecular subtyping of breast cancer is mainly based on transcriptome data (Xiong et al., 2025). Compared to transcriptome data, which can be affected by cell type, tissue origin, and sample processing, somatic mutation data are relatively stable (Kerle et al., 2025), and could reveal how genetic mutations affect the role of tumor ecosystem in breast cancer, rather than simply highlighting gene expression patterns (Xie et al., 2025).

In this study, the prognostically associated subtypes of breast cancer were identified using somatic mutation profiles of 2,526 breast cancer patients obtained from MSKCC and network propagation algorithms. To address the sparsity and binary nature of the raw somatic mutation matrix, which limits direct statistical analysis and clustering, we employed a network propagation algorithm based on RWR. The mutation spectrum is then smoothed using the STRING protein-protein interaction network and assigned an appropriate value to each node, in which the mutant gene obtains a higher value. This approach propagates mutation information through the protein-protein interaction network, converting binary mutation data into continuous values, incorporating biological context from gene functional relationships, and improving statistical power for downstream analyses such as ssGSEA and clustering, thereby effectively reducing sparsity and improving data stability. 68 tumor ecosystem genesets in each patient were then quantified as NESs. Based on the NESs, breast cancer patients were separated into two subtypes by K-means clustering, and it was found that these two subtypes had significant differences in clinical marker, survival prognosis, immune microenvironment, and treatment response.

To our knowledge, this study is the first to stratify MSKCC breast cancer patients based on somatic mutation data, combined with network propagation algorithm and tumor heterogeneity analysis, providing a novel subtyping strategy for the study of the molecular mechanism of breast cancer (Su et al., 2026). The validation results show that our model has good performance, which may be related to the following potential reasons. Firstly, this study analyzed somatic mutation data to effectively avoid the influence of microenvironment and batch effect on gene expression level (Tomlinson, 2001). Moreover, somatic mutation data can directly reflect the genetic characteristics and driving mechanism of breast cancer, which makes the typing results of this study more reasonable and has good generalization ability. Secondly, breast cancer patients were categorized according to their tumor ecosystem. The tumor ecosystem landscape has been demonstrated to exert a pivotal influence on the progression of cancer and the shaping of the immune microenvironment. Our study demonstrated that tumor ecosystem subtyping was significantly associated with patient survival, immune characteristics, and treatment response. Thirdly, conventional breast cancer subtyping methods, such as molecular subtyping, are usually applicable to breast cancer itself, while the method in this study still demonstrates a robust capacity for classification in a range of other cancer subtypes and possesses significant versatility. In conclusion, our study underlines the possibility of classifying breast cancer patients based on their somatic profile, thus providing new insights for personalized treatment and prognosis of breast cancer patients.

However, several limitations ought to be pointed out. Firstly, our data source is derived from the MSKCC cohort and American patients, lack of breast cancer samples from other countries or origins. In future studies, multi-center data can be combined to verify the applicability of our classification scheme. We performed univariate Cox regression filtering prior to K-means clustering to reduce dimensionality, remove noise from prognostically irrelevant gene sets, and enhance survival-related signals. A P-value threshold of <0.05 was selected based on conventional statistical practice. We acknowledge that this pre-filtering may introduce bias by potentially excluding gene sets with small but biologically meaningful effects, and we also note that other thresholds (e.g., P-value <0.01 or P-value <0.1) were not tested, which represents a limitation of the current study. Secondly, although somatic profile discloses the prime mutant agent of breast cancer, the biological behavior of oncology is also impacted by other factors, such as transcriptional regulation, epigenetic modification, non-coding RNA, etc. Therefore, the incorporation of multiple omics studies, like epigenomic data, transcriptional data may prove to be more optimal. Thirdly, our study solely utilized the RWR algorithm and the STRING network to smooth the sparse somatic profile. The STRING database (version 11.5) was selected due to its comprehensive integration of experimentally validated and predicted protein-protein interactions, making it a widely used and reliable resource in network biology. An interaction score threshold of 700 (corresponding to “high confidence”) was chosen to ensure high-confidence interactions, which helps reduce noise from spurious or low-confidence edges. We acknowledge that other PPI networks (e.g., BioGRID, HINT, InWeb_IM) were not compared, and exploring their performance represents a potential direction for future research. The performance of other different algorithms can be further compared, such as Heat Diffusion, Page Rank, etc., to select the optimal algorithm or integrate multiple algorithms. Regarding the XGBoost classifier, hyperparameters were optimized using grid search combined with 10-fold cross-validation on the training cohort. The search space included learning_rate (0.1, 0.01, 0.001), n_estimators (50, 100, 200), max_depth (3, 6, 9), and colsample_bytree (0.6, 0.8, 1.0). The final optimized hyperparameters were: learning_rate = 0.1, n_estimators = 100, max_depth = 3, and colsample_bytree = 0.8, with objective = binary:logistic, and random_state = 123. We acknowledge that parameters including subsample, gamma, reg_alpha, and reg_lambda were kept at XGBoost default values and were not tuned, which represents a technical limitation that could be addressed in future studies through more comprehensive hyperparameter optimization.

To translate the proposed TES strategy into a potential clinical workflow, the following step-by-step process could be envisioned: (1) obtain somatic mutation profiles from tumor sequencing of a breast cancer patient; (2) apply the RWR-based network propagation algorithm to reduce data sparsity and generate continuous mutation profiles; (3) calculate normalized enrichment scores (NES) for the 68 tumor ecosystem gene sets using ssGSEA; and (4) input these NES values into the pre-trained XGBoost classifier to assign the patient to TES 1 or TES 2. TES 1 is associated with better prognosis, higher immune infiltration, and greater immunotherapy response, while TES 2 shows poorer prognosis, lower immune activity, and higher sensitivity to certain chemotherapeutic agents. This classification could thus guide clinical decisions regarding prognosis stratification, immunotherapy eligibility, and chemotherapy selection.

In summary, while this study has several limitations, including potential data bias due to the single-cohort (MSKCC) and US-only patient population, as well as limited generalizability to certain cancer types where significant prognostic separation was not observed, future directions including multi-omics integration, multi-center validation, and exploration of alternative algorithms and thresholds will further strengthen the applicability and robustness of our TES framework.

## 5 Conclusion

Overall, our study may provide a stable and reproducible breast cancer subtyping strategy for patients with somatic mutation data using MSKCC and may be generalized to a wide range of other cancers, potentially providing new ideas for personalized cancer treatment. Moreover, the performance and generalization ability can be further enhanced by combining multi-center data and multi-omics analysis.

## Data Availability

The original contributions presented in the study are publicly available at: https://www.cbioportal.org/study/summary?id=msk_met_2021. The source code and some raw experimental data used in this study is available at the following GitHub repository: https://github.com/LeiyangHarbin/frontier-in-genetics.
