# Supplementary material for: Tumor ecosystem subtyping of breast cancer based on somatic mutations and network propagation reveals distinct prognostic and genomic landscapes
Source: Front Genet. 2026 May 15;17:1847952. doi: 10.3389/fgene.2026.1847952 (PMC13218690; doi:10.3389/fgene.2026.1847952)
Supplement: Supplementary file 1 [file DataSheet1.pdf]

Supplementary Figures

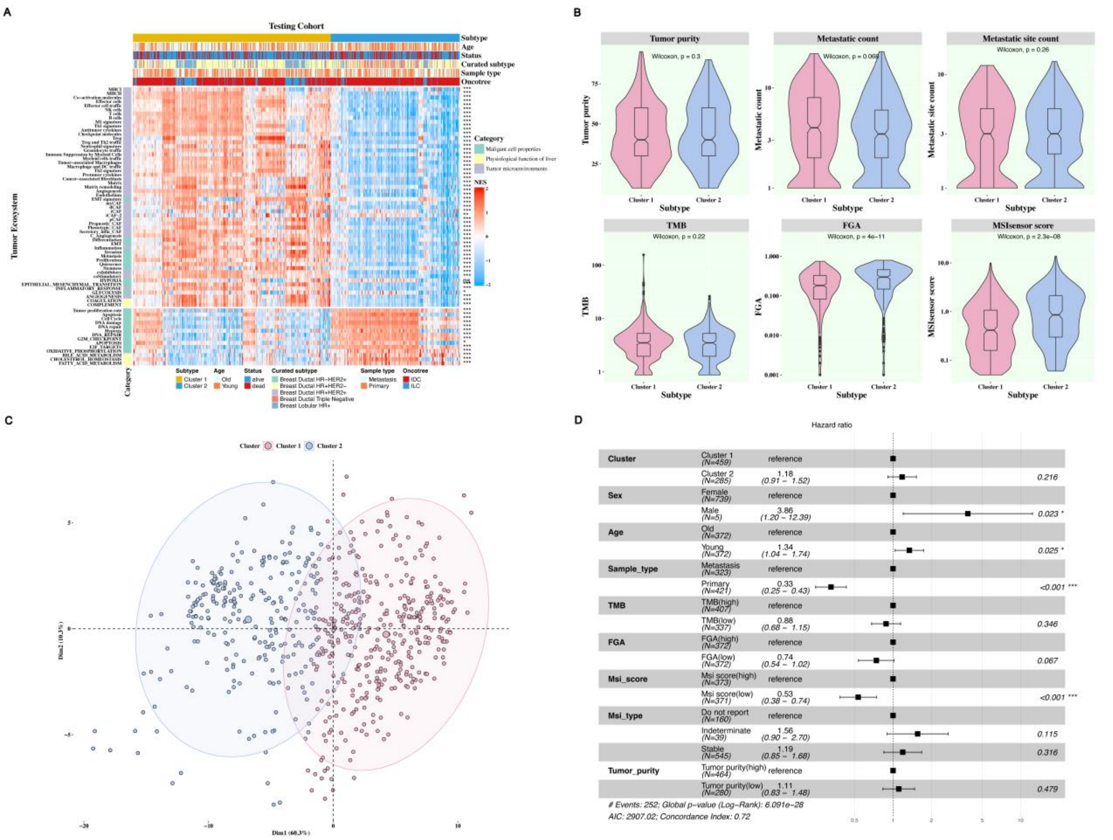

Fig. S1 The clinical signature and functional characteristics of the two cluster subtypes in the breast cancer testing cohort.

(A) The distribution of activity levels across 68 genesets discerning variations between the two cluster subtypes in the testing cohort. Each column represented one breast cancer sample, and each row represented a hallmark. The subtype, age, status, curated subtype, sample type and oncotree for each sample were marked on the top panel with different colors. Old age and young age were split by the median age (59.57 years old) in 2526 MSKCC breast cancer patients. (ns indicated P-value>0.05; indicated *P-value<0.05; **indicated P-value<0.01; ***indicated P-value<0.001; Wilcoxon rank-sum test)

(B) The violin plots distribution of tumor purity, metastatic count, metastatic site count, TMB, FGA and MSIsensor score between two cluster subtypes in testing cohort. (Wilcoxon rank-sum test)

(C) The PCA analysis projected breast cancer samples of testing cohort onto

two-dimensional spatial coordinates, demonstrating good discrimination for two cluster subtypes.

(D) A forest plot of multivariate Cox regression analysis of including the cluster subtypes and clinical annotations for breast cancer patients in testing cohort.

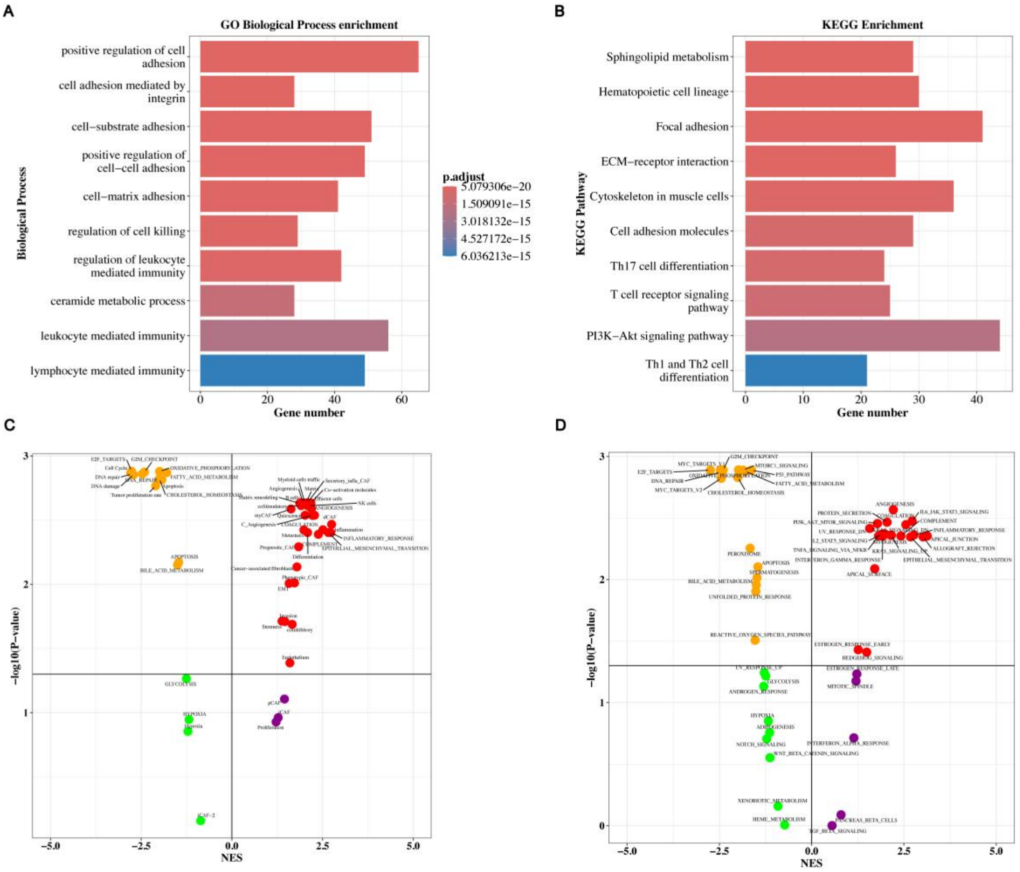

Fig. S2 Enrichment analysis result of two TESs in testing cohort.

(A) GO biological process terms and **(B)** KEGG pathways for the differential genes between two clustering subtypes in testing cohort.

(C) Volcano plot illustrated the GSEA result of 68 genesets and **(D)** 50 Hallmarks between two clustering subtypes in testing cohort. The enriched signatures were marked with orange color. The x-axis was utilized to display NES, while the y-axis was utilized to display -log10 (P-value).

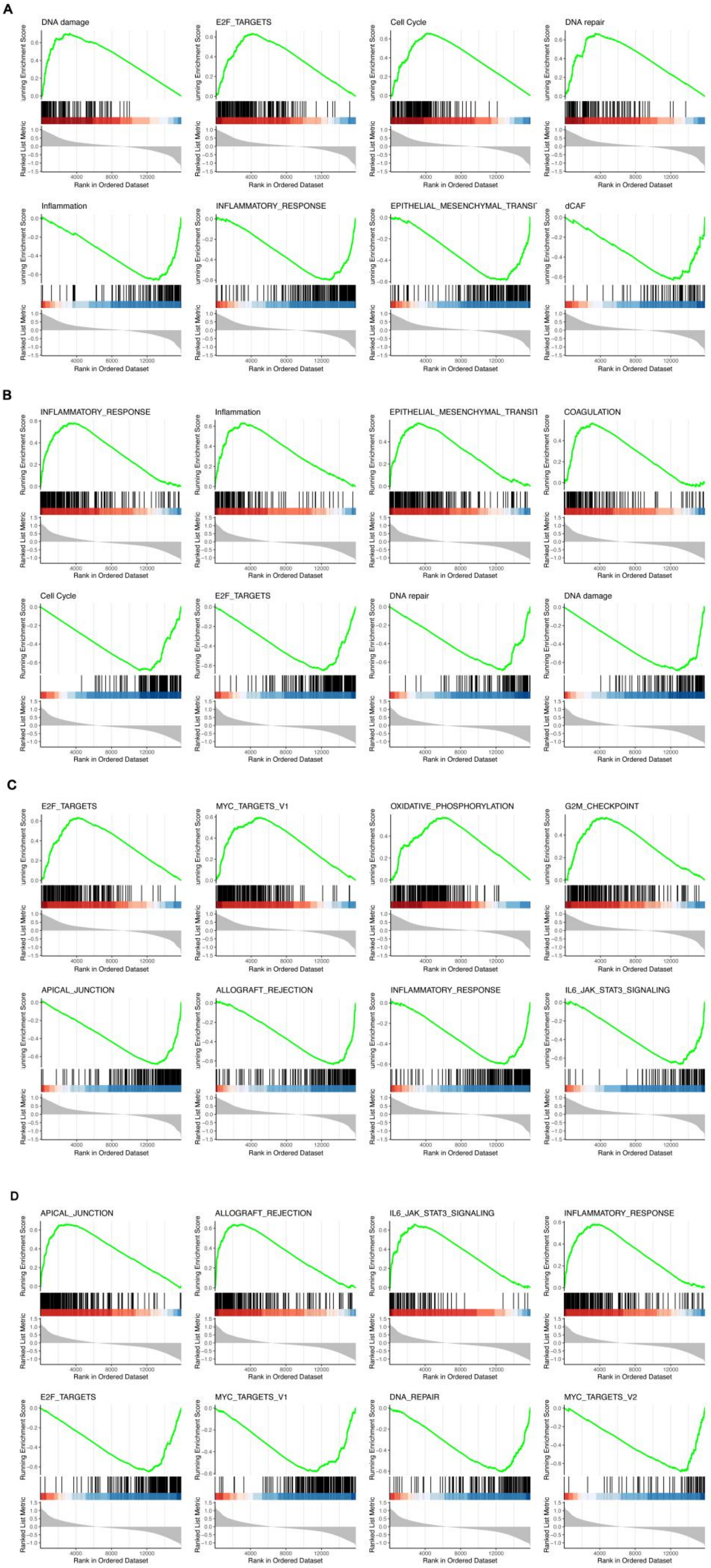
A
DNA damage
E2F_TARGETS
Cell Cycle
DNA repair
Inflammation
INFLAMMATORY_RESPONSE
EPITHELIAL_MESENCHYMAL_TRANSI
dCAF
B
INFLAMMATORY_RESPONSE
Inflammation
EPITHELIAL_MESENCHYMAL_TRANSI
COAGULATION
Cell Cycle
E2F_TARGETS
DNA repair
DNA damage
C
E2F_TARGETS
MYC_TARGETS_V1
OXIDATIVE_PHOSPHORYLATION
G2M_CHECKPOINT
APICAL_JUNCTION
ALLOGRAFT_REJECTION
INFLAMMATORY_RESPONSE
IL6_JAK_STAT3_SIGNALING
D
APICAL_JUNCTION
ALLOGRAFT_REJECTION
IL6_JAK_STAT3_SIGNALING
INFLAMMATORY_RESPONSE
E2F_TARGETS
MYC_TARGETS_V1
DNA_REPAIR
MYC_TARGETS_V2
Running Enrichment Score
Ranked List Metric
Rank in Ordered Dataset

Fig. S3 GSEA analysis of two cluster subtypes in the breast cancer training and testing cohort.

GSEA plots were generated to visualize the enrichment results of the most significant 4 pathways at the top and bottom of the NES gene set in the respectively **(A)** training and **(B)** testing cohorts as well as the hallmark gene set in the corresponding **(C)** training cohort and the **(D)** testing cohorts between two clusters.

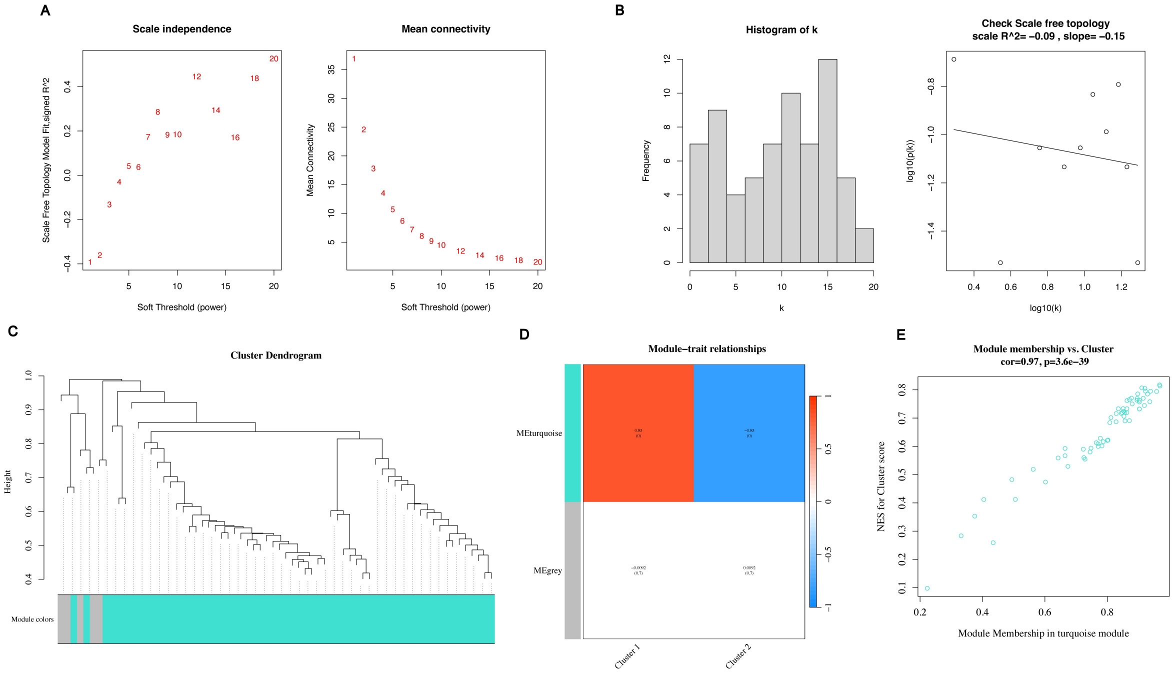

Fig. S4 Determination of soft-thresholding power in the gene weighted co-expression network analysis.

(A) The soft threshold power of 6 was selected to ensure a scale-free network. When the soft threshold was set to 6, the constructed network conformed to the power-law distribution and was closer to the real biological network state.

(B) The connectivity histogram and the scale-free topology test when β=6.

(C) The hierarchical clustering tree diagram identified two modules.

(D) Correlation analysis between module eigengenes and cluster traits.

(E) Scatterplot of gene significance (GS) for NES level versus module membership (MM) in the turquoise module.

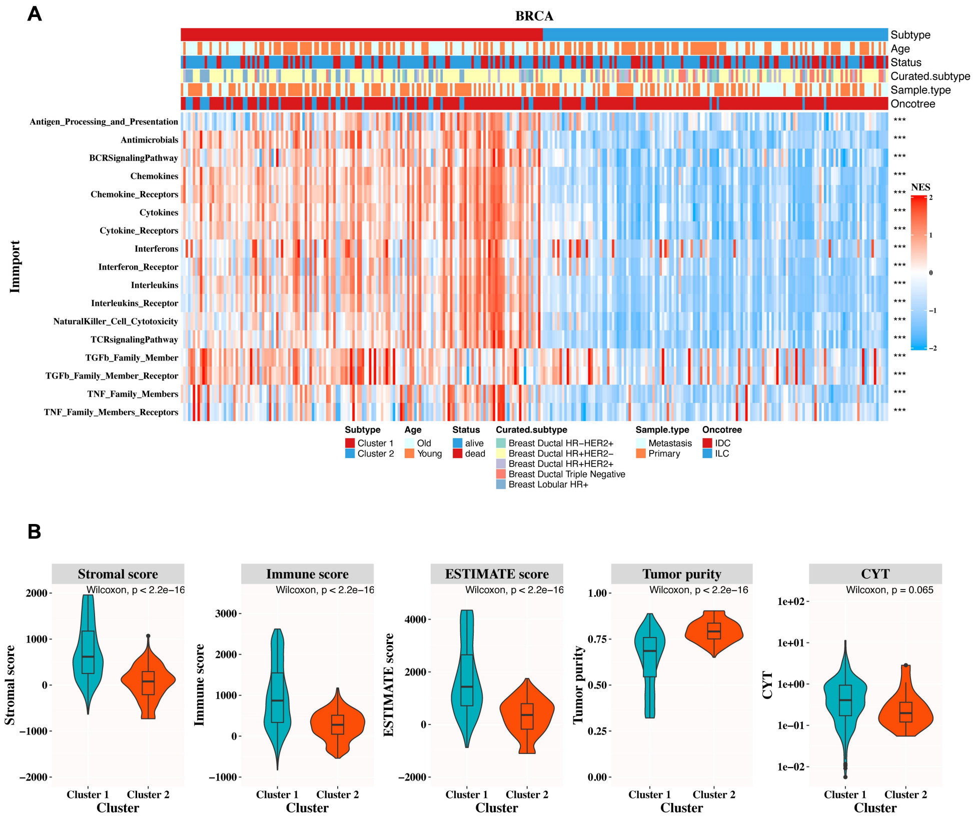

Fig. S5 Immunological research of two different TESs in the testing cohort.

(A) The NES levels of 72 immune checkpoints in two distinct breast cancer clustering subtypes in testing cohort. Old age and young age were split by the median age (59.57 years old) in the 2526 MSKCC breast cancer patients. ICP indicated immune checkpoint; ns indicated P-value>0.05; * indicated P-value<0.05; *** indicated P-value<0.001; Wilcoxon rank-sum test.

(B) Violin plots illustrated the differences in Stromal score, Immune score, ESTIMATE score, Tumor purity, and CYT of two clustering subtypes in testing cohort. According the Wilcoxon rank-sum test, significant differences were found in Tumor purity, ESTIMATE score, Stromal score, and Immune score between two clustering subtypes.

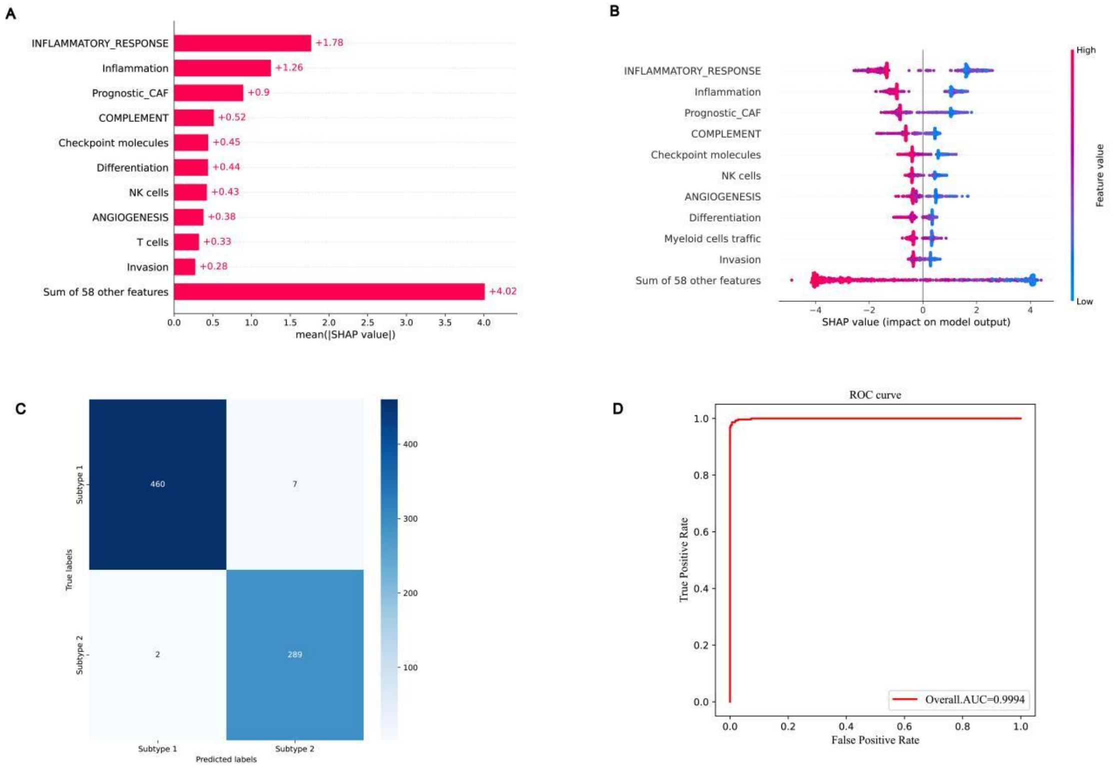

Fig. S6 Prediction of breast cancer patients with different TESs by XGBoost algorithm in the testing cohort.

(A) The mean absolute SHAP values of the top 10 genesets.

(B) Distribution of the SHAP values for the top 10 genesets based on the highest mean absolute SHAP value. The x-axis illustrated the SHAP value. The colors of the dots corresponded to the magnitude of their respective observed values for genesets, ranging from small to large or negative to positive.

(C) Confusion matrix for prediction of breast cancer patients in the testing cohort.

(D) The ROC curve of XGBoost model for prediction of breast cancer patients in the testing cohort.
